# Risk for Severe Group A Streptococcal Disease among Patients’ Household Contacts

**DOI:** 10.3201/eid0904.020369

**Published:** 2003-04

**Authors:** Katherine A. Robinson, Gretchen Rothrock, Quyen Phan, Brenda Sayler, Karen Stefonek, Chris Van Beneden, Orin S. Levine, for the Active Bacterial Core Surveillance/Emerging Infections Program Network

**Affiliations:** * Centers for Disease Control and Prevention, Atlanta, Georgia, USA; † California Department of Health Services, Oakland, California, USA; ‡ University of California, Berkeley, California, USA; § Connecticut Department of Public Health, Hartford, Connecticut, USA; Minnesota Department of Health, Minneapolis, Minnesota, USA; # Oregon Department of Human Resources, Portland, Oregon, USA

## Abstract

From January 1997 to April 1999, we determined attack rates for cases of invasive group A streptococcal (GAS) disease in household contacts of index patients using data from Active Bacterial Core Surveillance sites. Of 680 eligible index-patient households, 525 (77.2%) were enrolled in surveillance. Of 1,514 household contacts surveyed, 127 (8.4%) sought medical care, 24 (1.6%) required hospital care, and none died during the 30-day reference period. One confirmed GAS case in a household contact was reported (attack rate, 66.1/100,000 household contacts). One household contact had severe GAS-compatible illness without confirmed etiology. Our study suggests that subsequent cases of invasive GAS disease can occur, albeit rarely. The risk estimate from this study is important for developing recommendations on the use of chemoprophylaxis for household contacts of persons with invasive GAS disease.

Group A streptococcus (GAS) causes a wide range of illnesses from noninvasive disease such as pharyngitis and pyoderma (*1*,*2*) to more severe invasive infections (e.g., bacteremia, pneumonia, and puerperal sepsis) (*3*,*4*). In the 1980s, invasive GAS infections received increasing attention from the medical community and the public because of necrotizing fasciitis (NF) (*5*,*6*) and the emergence of streptococcal toxic shock syndrome (STSS) (*7*–*10*). Based on results of the Active Bacterial Core Surveillance (ABCs)/Emerging Infections Program network, a population-based surveillance system, the Centers for Disease Control and Prevention (CDC) estimates that, in 1999, the annual invasive GAS incidence was 3.5 cases per 100,000 population, yielding approximately 9,400 cases and 1,200 deaths in the United States each year (*11*).

The severity of GAS disease, coupled with a number of case clusters reported in communities and families (*12*–*14*) and several anecdotal reports of subsequent cases of invasive GAS infection in close contacts, causes concerns about the spread of disease among close contacts and questions about whether chemoprophylaxis to prevent illness in close contacts is warranted. Using data from active surveillance in Ontario, Canada, where the baseline rate of sporadic invasive GAS disease was 2.4 per 100,000 population (pers. comm.), investigators estimated that the attack rate of disease among household contacts of patients with invasive GAS disease was higher than the rate of invasive disease among the general population (294.1/ 100,000 population) (*3*).

In October 1995, the Working Group on Prevention of Invasive GAS Infections, composed of streptococcal experts from a variety of clinical and public health organizations, CDC, and various academic institutions, held a meeting to examine existing data and to determine if these data were sufficient to recommend widespread use of chemoprophylaxis to prevent subsequent invasive GAS disease among close contacts of index patients. Four specific criteria were used (*15*): severity of disease (*16*–*19*), virulence of the strain (*18*,*20*–*23*), increased risk for subsequent disease, and availability of an effective chemoprophylaxis regimen. Both the severity of invasive GAS disease and the virulence of GAS strains had been well documented. However, at that time, limited data existed regarding the risk for subsequent GAS disease among household contacts and an optimal regimen for chemoprophylaxis.

The working group concluded that a single study with four case-pairs was inadequate for establishing national recommendations for chemoprophylaxis for subsequent invasive GAS illness and emphasized the need for additional data on the risk of subsequent GAS disease among household contacts (*15*). We conducted surveillance to quantify the subsequent attack rates for both confirmed invasive GAS disease and severe GAS-compatible disease with no known etiology among household contacts in four geographic areas in the United States.

## Methods

### Identification of Index Patients

Cases of invasive disease attributed to GAS were identified through ABCs from January 1, 1997, to April 30, 1999. Active, population-based surveillance for laboratory-confirmed GAS infections occurred in four areas: the states of Connecticut and Minnesota; the San Francisco Bay area, California (three counties); and Portland, Oregon, (three counties). The aggregate population in 1998 was 12.1 million, or 4.5% of the U.S. population.

Invasive GAS disease was defined as the isolation of *Streptococcus pyogenes* in a surveillance area resident from a normally sterile site (e.g., blood or cerebrospinal fluid) or from a wound (when accompanied by STSS or NF). Surveillance personnel reviewed records of all 208 clinical laboratories in the participating ABCs areas every 6 months to verify completion of case ascertainment. All available sterile site isolates were sent to CDC for confirmation and further microbiologic testing (e.g., *emm*-typing) (*24*).

A GAS index patient was defined as the person with the first invasive GAS infection in a household. A nosocomial GAS case was defined as a case-patient with a date of first positive culture obtained >2 days after admission to hospital. An institutional GAS case was defined as a case-patient who resided in a nursing home, jail, long-term skilled-care facility, or other long-term care institution.

### Identification of Eligible Households of Index Patients

Surveillance personnel contacted the households of all index patients to determine study eligibility. We restricted eligibility to households of index patients with community-acquired GAS infections. We excluded households of nosocomial, institutionalized, and homeless GAS index patients in addition to households of index patients who lived alone or were without phones. To reduce the effect of recall bias, we excluded households from which the case was not identified within 120 days of the culture date.

### Collection of Information on Household Contacts

We defined a household contact as a person who regularly spent 50% of nights or >24 h in a household with the index patient during the week before the index patient’s date of culture. The index patients (or appropriate adult surrogates) of eligible households were interviewed by telephone within 31 to 120 days after the index patient’s date of culture. Information collected on all household contacts included age, gender, underlying conditions, and relationship to the index patient. Study personnel also identified all household contacts who had sought medical care for any reason, been hospitalized, or died during the reference period.

Surveillance personnel abstracted the medical charts of all household contacts who had sought medical care, using a standardized data collection form to determine the types of visits, chief complaints, diagnostic tests results, type and duration of antibiotic use, and discharge diagnoses. All available sterile site GAS isolates from household contacts were collected and sent to CDC for confirmation and molecular testing.

We defined the study reference period as the 30 days after the index patient’s date of GAS culture. A confirmed case of subsequent invasive GAS disease was defined as isolation of GAS from a household contact collected from a normally sterile site (or from a wound when accompanied by NF or STSS) within the study reference period. A probable case of subsequent severe disease was defined as a GAS-compatible illness resulting in hypotension, hospitalization, or death within the study reference period in a person from whom GAS was not isolated and for whom other infectious causes of disease were ruled out.

### Analysis

Analysis was performed by using SAS software, version 6.12 (SAS Institute, Inc., Cary, NC) and Epi Info, version 6.04c (CDC, Atlanta, GA). Attack rates (number of subsequent cases of invasive or severe GAS disease divided by number of household contacts, expressed as subsequent cases per 100,000 household contacts) were calculated for subsequent GAS disease among household contacts. We then compared the attack rate using only confirmed subsequent cases of invasive GAS disease to the sporadic incidence rate for invasive GAS disease among the general population to determine the increase in risk for subsequent GAS disease among household contacts. Exact 95% confidence intervals for the risk for subsequent GAS disease among household contacts were determined by using binomial distribution.

## Results

During the study period, 1,063 index patients with invasive GAS disease were identified, ranging in age from <1 year to 99 years (median, 48 years of age). The elderly (age >65 years of age) accounted for nearly one third (31.4%) of the invasive GAS cases. Most index patients had cellulitis with bacteremia (36.8%) or bacteremia with no focal point of infection (25.9%). Thirteen percent of the index patients had NF (6.5%), STSS (4.6%), or both (2.0%). Diabetes mellitus and alcohol abuse were the two most frequent medical conditions among patients with invasive GAS disease. Less than 5% of the index patients were infected with HIV.

Of the 1,063 households with index patients, 680 (64.0%) were eligible for the study. Ineligible households included those with index patients who had institutional infections (n=106, 10.0%), lived alone (n=106, 10%), had no telephone (n=42, 4.0%), or had nosocomial infections (n=37, 3.5%). Fifty-two (4.9%) of the index patients were homeless. Some households (n=36, 3.4%) were not eligible because the case was identified >120 days after the culture date. Of the 680 eligible index-patient households, 525 (77.2%) were enrolled. Eligible households not enrolled included those that could not be contacted (n=120, 17.6%) and those that refused to participate (n=24, 3.5%). Eleven households (1.6%) were not enrolled because of other reasons, primarily language barriers.

From the 525 enrolled households, 1,514 household contacts were identified and investigated (Table 1). Over half of the contacts were female (54%). The age distribution among the contacts was <93 years of age (median age, 29 years); 38.7% of contacts were children <18 years of age. Twelve percent of the household contacts (n=181) reported antibiotic use during the reference period. Approximately 9% (n=130) of the household contacts reported at least one underlying medical condition; the most common were chronic lung disease (3.0%) and congestive heart failure (2.6%).

**Table 1:** Demographic and clinical features of household contacts of invasive group A streptococcus index patients^a^

Demographic or clinical feature	No. of household contacts^b^	Proportion of all household contacts (%)
Age (in y)^c^		
0–4	177	11.9
5–17	398	26.7
18–34	324	21.7
35–49	290	19.5
50–64	157	10.5
>65	145	9.7
Sex^d^		
Male	697	46
Female	810	54
Underlying medical condition		
Chronic lung disease	46	3.0
Congestive heart failure	39	2.6
Insulin-dependent diabetes	32	2.1
Cancer (except skin)	23	1.5
Other immunocompromising conditions^e^	18	1.2
Liver disease	11	0.7
Chronic kidney disease	6	0.4

^a^N=1,514. ^b^Household contacts are counted more than once if multiple conditions exist. ^c^Age was missing for 23 household contacts. ^d^Sex was unknown for seven household contacts. ^e^Includes HIV infection, AIDS, intravenous drug use, chemotherapy for cancer, steroid use for other conditions such as recent organ transplant, or any illness from excessive use of alcohol.

Of the 1,514 household contacts, 127 (8.3%) sought medical care or were hospitalized during the reference period. No household contacts died during the reference period. Of the 127 household contacts who visited a physician, 104 (81.9%) reported having symptoms; however, 23 (18.1%) were asymptomatic at the time of their visit. Twenty of the asymptomatic household contacts reported visiting the physician because a family member had been ill with invasive GAS infection. Of the 104 symptomatic household contacts, infectious illness was diagnosed in 62 (59.6%). The diagnosis for most of these contacts was based on clinical evidence of streptococcal pharyngitis (n=10), obtained with a positive rapid strep test (n=36) or a positive throat culture (n=5). Eight cutaneous infections, one case of pneumonia documented by x-ray with no positive culture, and two clinically diagnosed cases of pneumonia were diagnosed in contacts. Of the 23 asymptomatic household contacts, 15 (65.2%) had evidence of GAS in the throat from a rapid strep test (n=13) or positive throat culture (n=2). Twenty-four household contacts required hospital care for various reasons during the reference period (13 hospital admissions and 11 emergency room visits).

During the study period, we identified one confirmed subsequent case of invasive GAS disease and one probable subsequent severe GAS disease in household contacts (Table 2). Both cases were diagnosed in immediate family members and resulted in hospitalization. The index patient in the one confirmed case-pair was a 76-year-old woman who was hospitalized with cellulitis and had a positive blood culture for GAS. The contact was her 69-year-old husband, who was hospitalized with cellulitis that progressed to NF 15 days after the index patient’s culture date. A surgical specimen grew GAS, but the isolate was not available for confirmation or further testing by CDC. Both patients had underlying medical conditions.

**Table 2:** Confirmed and probable subsequent invasive group A streptococcus disease case-pairs, Active Bacterial Core Surveillance (ABCs)^a^

Case-pair status	Case status	ABCs area	Sex	Age (in y)	Interval (d)	Diagnosis	GAS culture results	Underlying condition	Hospitalized?	
Confirmed	Index	CA	Female	76	—	Cellulitis	Blood +	COPD, CHF	Yes	
	Household Contact		Male	69	15	Necrotizing fasciitis	Tissue + Blood –	Venous insufficiency	Yes	
Probable	Index	CT	Female	0	—	Bacteremia	Blood +	None	Yes	
	Household contact		Male	39	19	Erysipelas	Blood –	None	Yes	

^a^GAS, group A streptococcus; Active Bacterial Core Surveillance (ABCs); CA, California; CT, Connecticut; +, positive; –, negative; COPD, chronic obstructive pulmonary disease; CHF, congestive heart failure.

The probable case-pair included an infant daughter and her father. The index patient was a 2-month-old girl hospitalized with GAS bacteremia with no focal point of infection. Her 39-year-old father was hospitalized 19 days after his daughter’s date of culture; he had erysipelas accompanied by fever and hypotension (systolic blood pressure 86 mM Hg); a single blood culture was negative for GAS. He was hospitalized for 2 days and given intravenous antibiotics at home for 14 days. Neither the infant nor her father had underlying medical conditions.

We compared the attack rates of subsequent GAS disease in household contacts for this study to the Ontario, Canada, study (*3*). The attack rate of our study, using only confirmed cases of subsequent disease from ABCs, was 66.1 per 100,000 household contacts (95% confidence intervals [CI] 2 to 367). When both confirmed and probable cases of subsequent disease were used, the attack rate was 132.1 per 100,000 household contacts (95% CI 16 to 476); an estimate that remains lower than that measured among the Canadian study population.

## Discussion

During the 2-year study period in a population of 12.1 million, we identified one confirmed subsequent case of invasive GAS disease, resulting in an estimated risk of 66.1 per 100,000 household contacts. This attack rate represents an increased risk for disease among household contacts of index patients when compared to the annual incidence rate of sporadic invasive GAS disease in the United States (average rate 3.5/100,000 population, 1995–1999) (*16*). Although the risk estimate from this study is lower than the risk previously reported from surveillance in Canada, both risk estimates have extremely wide confidence intervals.

Our study has several strengths, including the large defined population base in four geographically diverse regions in the United States that participated in laboratory-based surveillance. The methods and completeness of case ascertainment of invasive infections for the ABCs system are well established. Also, the charts of all household contacts who reported seeking medical care during the 30-day reference period were reviewed for invasive or severe GAS infections.

The baseline rate of sporadic invasive GAS disease in this U.S. study was higher than that observed in the Canadian population, while the risk for subsequent GAS disease was lower than found in the Toronto study. Given the wide confidence intervals, a comparison of the risk estimate of subsequent infections between the two studies is not warranted. Further complicating a comparison of the studies are differences in physician management and frequency of blood culturing, factors that may affect the reported rate of sporadic invasive GAS disease.

Our study was limited in the lack of information on the use of chemoprophylaxis. We did not directly ask the household contacts or the physicians about the use of prophylactic antibiotics. Thus, we were unable to consistently determine the number of household contacts who received prophylactic antibiotics specifically for the prevention of GAS disease from their physicians during the reference period. Another limitation of the study is related to the reasons why household contacts sought medical care. Although the chart abstraction form asked about chief complaints, it did not specifically ask if the contacts were asymptomatic and sought medical care simply because a family member had been ill with invasive GAS. We were therefore unable to consistently differentiate between household contacts who sought medical care for actual symptoms or illness from those who sought medical care simply because a family member had been ill from GAS.

Caution should be taken when defining the magnitude of increased risk for subsequent invasive GAS disease to household contacts compared with the risk for invasive GAS disease among the general population. The attack rates for confirmed and probable severe GAS disease in household contacts from this study are based on minuscule numbers (one and one, respectively), resulting in estimates with extremely wide confidence intervals. Even if the confirmed cases from this study and the Canadian study were combined, the point estimate would be based on five cases from 2,874 household contacts observed over several years of surveillance, and the confidence intervals would remain wide. Given that the combined population and duration of both studies are 22.8 million persons and 4.5 years, a well-designed prospective study would be necessary to achieve a risk estimate with narrower confidence intervals and is likely not feasible.

Additionally, while both studies show an increase in risk for subsequent disease among household contacts, directly comparing the risk to the incidence of primary invasive disease is problematic (*25*). The attack rate of household contacts was determined during a 30-day period as opposed to a year because any risk for subsequent disease would likely be concentrated in the period shortly after the occurrence of the index case in the household. We think the data are best interpreted as additional evidence that household members are at higher risk for invasive GAS disease during the month following the index patient’s illness than are others in the population but that the absolute risk for subsequent disease is low.

Because of the small numbers of case-pairs, predicting who is most likely to acquire a severe subsequent GAS infection is difficult based on either this study or the Canadian study. All five subsequent cases in the two reports occurred among adults who were immediate family members, and all five occurred within 3 weeks of the index patient’s date of culture. Although we cannot predict who will acquire an invasive GAS infection from a household member, multiple published studies have identified those persons who are more likely to acquire sporadic invasive GAS infections that are unrelated to contact with infected persons and those who are more likely to die from an invasive infection. Groups at increased risk for sporadic disease include those who have recently been infected with varicella-zoster virus; have HIV infection, diabetes, cancer, or heart disease; are currently using high-dose steroids or intravenous drugs; or are Native American. Persons >65 years of age are more likely to die following an invasive GAS infection than other age groups (*3*,*10*,*11*,*16*).

This study provides important information for healthcare practitioners and public health personnel to help guide their responses to invasive GAS cases. The results of this study and the Canadian study, the potential impact of chemoprophylaxis, data on possible effectiveness of chemoprophylactic regimens, and the overall epidemiology of invasive GAS infections were recently reviewed by the Prevention of Invasive Group A Streptococcal Infections Working Group. The group concluded that although the risk for subsequent invasive GAS disease in household contacts is higher than the risk among the general population, routine administration of chemoprophylaxis to all household contacts of persons with invasive disease is not recommended given the infrequency of these infections and the lack of a known effective chemoprophylactic regimen (*26*). Clinicians and public health professionals should inform household members of persons with invasive GAS infections about the early clinical manifestations of pharyngeal and invasive GAS disease.
